# Bronchial Carcinoma: A Statistical Study of 741 Necropsies with Special Reference to the Distribution of Blood-Borne Metastases

**DOI:** 10.1038/bjc.1955.53

**Published:** 1955-12

**Authors:** S. Galluzzi, P. M. Payne


### BRONCHIAL CARCINOMA: A STATISTICAL STUDY OF 741 NECROPSIES WITH SPECIAL REFERENCE TO THE DISTRIBUTION OF BLOOD-BORNE METASTASES.

S. GALLUZZI AND P. M. PAYNE

*From the Radiotherapy Department of the Royal Marsden Hospital, London, S.W.3.*

Received for publication October 18, 1955.

ADVANCES in treatment have made possible the local eradication of many primary malignant tumours; in contrast, little progress has been made with the much more difficult problems of influencing blood stream spread or of dealing effectively with established multiple metastases. Except for the temporary control by alteration in hormone balance of a few special types of tumour little of value to the patient has yet come from much detailed fundamental research work on the chemical control of cancer. It would seem, therefore, that one of the most important investigations in clinical cancer research at the present time concerns those factors which determine the production and localisation of metastases.

The reasons for the observed distributions of blood-borne metastases have been a matter of controversy for many years. At one extreme we have the view that cells from certain invasive tumours are commonly released into the blood stream at an early stage and that whether metastases then develop and what their number and distribution may be, depends on the nature of the tumour cells and the suitability of the tissues in which they lodge as an environment for this growth. At the other extreme it is held that the entry of tumour cells into the blood stream depends largely on differences in tissue tensions and that if they do get into the circulation, the distribution of the resulting metastases will depend on the size of the cell clumps, the relative sizes of the organs and the state of their capillaries. The first may be called the "seed and soil" theory and the second the "mechanical" theory. It is almost certain that the truth is a variable and complicated association of the two.

When animals have tumour suspensions injected into their arterial circulations, each tumour tends to produce its own metastatic pattern, though metastases are frequently found in some organs whatever tumour suspension is used (Sugarbaker, 1952). If care is taken to inject tumour suspensions simultaneously into the blood supply of the liver and the lung so that metastases become established at the same time in both, the liver tumours tend to grow faster than the lung tumours. This suggests that for these two organs and for some tumour types at least, tissue environment is an important factor (Lucke, Breedis, Woo, Berwick and Nowell, 1952).

It is only too obvious that some types of tumour metastasise more readily than others, but it is not so clear whether this property depends upon their inherent invasiveness, alterations in neighbouring tissue restraints, the tissue pressures exerted (Young and Griffith, 1950), or the elaboration by the tumour cells of enzymes which cause connective tissue destruction. It seems certain that there is much more tumour cell dissemination than metastasis and that many cells are destroyed in the blood stream. Tumour cells can be seen in lung capillaries (Willis, 1952) and in sternal bone marrow (Kreyberg and Poppe, 1940) ; they can pass immediately through the pulmonary circulation in rabbits and rats at least (Zeidman and Buss, 1952) and may be shunted from the systemic to the vertebral venous system (Batson, 1940, 1942 ; Anderson, 1951). It is quite possible that they may also travel by other by-passing mechanisms such as those which operate in shock (Trueta *et al.*, 1947). There seems to be evidence that any part of the body may be presented with tumour cells from any primary site.

If we accept the hypothesis of general blood stream dissemination, the mechanical factors determining the distribution of metastases may be : the size of the tumour fragments (Zeidman, McCutcheon and Coman, 1950), whether the tumour cells occur in clumps or singly (Watanabe, 1954), their number, the state of the capillaries through which they pass, and the rate of blood flow within them. There is in fact no close correlation between the number of tumour cells reaching an organ and the number of metastases, but there is an excellent correspondence between the number lodged in capillaries and the number of secondary deposits (Coman, de Long and McCutcheon, 1951). The various organs of the body in rats seem to offer little resistance to the growth of tumour implants (Baxter, Leupold-Loewenthal and Burton, personal communication). Some tumours grow readily even in spleen and muscle (de Long and Coman, 1950), a fact which supports the contention that much depends on the number of cells which become arrested in an organ, and on the ease with which they can escape from the blood vessels.

Primary tumours of the lung would seem to form a good group for this type of clinical study, since for each tumour the chance of even dissemination throughout the blood stream is likely to be greater than for most other sites. We have therefore analysed the distribution of metastases found at necropsy in eight London hospitals ; four teaching hospitals and four Regional Board hospitals, to see if we can by this means present any useful facts and draw any interesting conclusions which might help towards the elucidation of this most important aspect of the cancer problem.

*Source of the material*

Details of 741 patients, who died with carcinoma of the bronchus in the years 1948–52, have been collected from the records of eight London hospitals. All these patients were subjected to careful clinical study during the course of their disease and a complete necropsy was performed in all but 94 cases where no intracranial examination was carried out.

During the same period the total number of necropsies performed at the eight hospitals was 10,430. Table I shows how this number was made up and what proportion of all necropsies related to patients who had died from carcinoma of the bronchus. In the case of the Royal Marsden Hospital this proportion is rather high (12·9 per cent) no doubt due to the special nature of its work and to its close association with the Brompton Hospital for Diseases of the Chest. If we exclude the Royal Marsden Hospital, 6·8 per cent of all necropsies related to patients who had died from carcinoma of the bronchus.

This percentage agrees with the figure of 6·9 per cent found in the British Empire Cancer Campaign's (1950) survey of the records of several London hospitals for for the years 1945–49. It is, however, much higher than either the 2·3 per cent

TABLE I.—*Sources of Material and Proportions of All Necropsies Performed on Patients who Died from Bronchial Carcinoma.*

| Hospital. | All necropsies 1948–52. | Number referring to carcinoma bronchus. | Per cent carcinoma bronchus. | Number with full necropsy including intracranial. examination. |
|---|---|---|---|---|
| Hammersmith Hospital | 1975 | 156 | 7·9 | 122 |
| St. Mary Abbots Hospital | 2016 | 113 | 5·6 | 90 |
| Middlesex Hospital | 1466 | 87 | 5·9 | 58 |
| St. George's Hospital | 1365 | 87 | 6·4 | 84 |
| Archway Hospital | 1034 | 83 | 8·0 | 82 |
| St. Mary's Hospital, Islington | 1424 | 99 | 7·0 | 99 |
| Highgate Hospital | 652 | 52 | 8·0 | 51 |
| All hospitals except Royal Marsden Hospital | 9932 | 677 | 6·8 | 586 |
| Royal Marsden Hospital | 498 | 64 | 12·9 | 61 |
| All hospitals including Royal Marsden Hospital | 10,430 | 741 | 7·1 | 647 |
| Four Teaching Hospitals | 5304 | 394 | 7·4 | 325 |
| Four Regional Board Hospitals | 5126 | 347 | 6·8 | 322 |

found by Heady and Kennaway (1949) or a similar figure found by Steiner, Butt and Edmondson (1950).

The patients in this series died with the disease in varying stages of development. Many of the patients (64 per cent*) had received no specific treatment and the remainder had been treated by a variety of methods. While therapeutic intervention may have influenced the spread of malignant emboli, many of the comparatively small number of treated patients had obvious metastases before treatment started.

The analysis of data obtained in this way, by the retrospective examination of records from a variety of sources, is fraught with difficulties. It cannot be expected to replace or approach in accuracy a properly planned laboratory experiment or a well designed clinical investigation. Certain suggestions or hypotheses may, however, emerge which might later be tested by more direct and controlled methods.

*Features of the material*

Before considering that part of the data which relates particularly to metastases it is important to examine in more detail the constitution of the series as a whole with special reference to factors such as sex, age and histology. By attending especially to differences between hospitals we may by this means learn something of selection processes and individual pathological interpretations which, in turn, may be useful when we come to study the data relating to metastases.

(i) *Sex*.—Table II shows the material analysed according to sex and age for each hospital. The proportions of females range from 14 per cent at St. Mary

* 313 out of 487 patients in respect of whom we have a definite record. This does not include the treatment records of the three wings of the Whittington Hospital, namely, Archway, St. Mary's Islington, and Highgate.

TABLE II.—*Sex and Age Distribution of Material by Hospitals.*

| Hospital. | Sex. | Age (years). 20– | 30 | 40– | 50– | 60– | 70– | 80– | Not stated. | Total. | Per cent females. | Mean age (years). | S.E. of mean (years). |
|---|---|---|---|---|---|---|---|---|---|---|---|---|---|
| (*a*) Hammersmith Hospital | M | — | 5 | 21 | 40 | 52 | 9 | 1 | — | 128 | 18 | 58·5 | 0·82 |
| | F | — | 3 | 3 | 10 | 5 | 5 | 2 | — | 28 | | | |
| (*b*) St. Mary Abbots Hospital | M | — | 3 | 10 | 25 | 34 | 22 | 3 | — | 97 | 14 | 63·0 | 0·83 |
| | F | — | 1 | — | 3 | 6 | 3 | 3 | — | 16 | | | |
| (*c*) Middlesex Hospital | M | — | 2 | 6 | 32 | 24 | 3 | — | 1 | 68 | 22 | 56·6 | 0·97 |
| | F | — | 4 | 4 | 4 | 6 | 1 | — | — | 19 | | | |
| (*d*) St. George's Hospital | M | — | 1 | 18 | 25 | 25 | 4 | — | — | 73 | 16 | 56·8 | 1·03 |
| | F | — | 2 | 2 | 4 | 4 | 1 | 1 | — | 14 | | | |
| (*e*) Royal Marsden Hospital | M | 1 | 4 | 8 | 16 | 22 | 1 | — | — | 52 | 19 | 56·3 | 1·30 |
| | F | — | 1 | 3 | 1 | 6 | 1 | — | — | 12 | | | |
| (*f*) Archway Hospital | M | — | — | 5 | 20 | 29 | 16 | 1 | — | 71 | 14 | 63·1 | 0·94 |
| | F | — | — | 1 | 3 | 5 | 3 | — | — | 12 | | | |
| (*g*) St. Mary's Hospital, Islington | M | — | — | 2 | 20 | 38 | 16 | 2 | — | 78 | 21 | 64·3 | 0·83 |
| | F | — | 1 | 1 | 4 | 10 | 4 | 1 | — | 21 | | | |
| (*h*) Highgate Hospital | M | — | 1 | 2 | 8 | 18 | 11 | 4 | — | 44 | 15 | 66·9 | 1·53 |
| | F | — | — | — | — | 2 | 6 | — | — | 8 | | | |
| All hospitals | M | 1 | 16 | 72 | 186 | 242 | 82 | 11 | 1 | 611 | 17·5 | 60·4 | 0·40 |
| | F | — | 12 | 14 | 29 | 44 | 24 | 7 | — | 130 | | 60·8 | 1·12 |
| (*a*), (*c*), (*d*) and (*e*) together | M | 1 | 12 | 53 | 113 | 123 | 17 | 1 | 1 | 321 | 18·5 | 57·5 | 0·51 |
| | F | — | 10 | 12 | 19 | 21 | 8 | 3 | — | 73 | | 56·9 | 1·52 |
| (*b*), (*f*), (*g*) and (*h*) together | M | — | 4 | 19 | 73 | 119 | 65 | 10 | — | 290 | 16·4 | 63·7 | 0·63 |
| | F | — | 2 | 2 | 10 | 23 | 16 | 4 | — | 57 | | 65·7 | 1·39 |

Abbots to 22 per cent at the Middlesex Hospital, but the differences are not significant. The proportion of females in the whole series (17·5 per cent) compares well with 17·2 per cent, the proportion of females among all deaths from carcinoma of the lung in the Administrative County of London during the same period (Registrar General, 1950, 1951, 1952, 1953).

(ii) *Age*.—In this respect the hospitals are divisible into two distinct groups. In the first, the teaching hospital group, the average age of the patients at necropsy was less than 60 years (generally about 57 years). In the second, the Regional Board hospital group, the average age exceeded 60 years (generally 63 or 64 years). The mean age of all the patients was 60·5 years, but this figure alone is not of much importance in view of the above heterogeneity. Table II shows the age distribution of the patients for the various hospitals together with the mean ages and the standard errors of these mean values.

It is of interest to compare the age distributions for the two groups of hospitals referred to above with the age distribution for deaths in England and Wales during 1948–52 (Registrar General, 1950, 1951, 1952, 1953). This is done separately for males and females in Fig. 1. It will be seen from this that the two groups of hospitals show exactly opposite features in this respect. The teaching group with the low average age at death has an excess of young and middle-aged people in both sexes, but a deficiency of people over seventy years. In the Regional Board group, with high average ages, there is a relatively lower proportion of persons under sixty.

Fig. 1.—Proportionate distributions of ages at death compared for necropsies at two groups of hospitals and for all deaths from cancer of the lung in England and Wales.

Fig. 2.—Proportionate distributions according to histology for each hospital.

(iii) *Histology*.—In considering histology we are faced with a new source of heterogeneity, namely, that arising from the individual interpretation of sections by different pathologists. Fig. 2 shows how the patients in the series were assigned to three main groups ; undifferentiated carcinoma, adenocarcinoma and squamous celled carcinoma. In the term " undifferentiated " we have included the following :

(*a*) " oat celled carcinoma ",
(*b*) " round celled carcinoma ",
(*c*) " polygonal celled carcinoma ",
(*d*) " anaplastic carcinoma " ; while " adenocarcinoma " includes those reported both as " adenocarcinoma " and as " columnar celled carcinoma ".

It will be seen that the undifferentiated group is by far the largest, making up 63·5 per cent of the whole series. Adenocarcinomas and squamous celled carcinomas account for 12·6 per cent and 23·9 per cent respectively. With regard to differences between hospitals, we find that the proportion assigned to squamous celled carcinoma ranges from 19 per cent at Highgate Hospital to 27 per cent at the Royal Marsden Hospital, though these differences are not significant. This is not the case when we come to the question of assessing differentiation between undifferentiated tumours and adenocarcinomas. Here the differences are highly significant and are almost certainly related to lack of uniformity in interpretation rather than to processes of selection. Taking undifferentiated tumours and adenocarcinomas together we find that the proportion of these which are designated as adenocarcinomas varies from 6 per cent at the Royal Marsden Hospital to 31 per cent at St. Mary Abbots Hospital.

(iv) *Age and sex.*—It is not proposed to discuss here how the age distribution of the series varies in the two sexes from one hospital to another. We may, however, examine whether there are any differences between the two groups of hospitals referred to in (ii). The age distribution of these two groups according to sex is set out at the bottom of Table II.

A study of these data reveals the following features :

(*a*) In the teaching group there is a marked difference in the way in which male and female ages are distributed, although the average ages do not differ much. The ratio of males to females reaches a maximum at about sixty but becomes less towards the extremes.

(*b*) In the Regional Board group there is some indication that the average age of females is greater, though the differences in the manner of distribution of male and female ages is much less marked.

(v) *Sex and histology.*—In the present series, 89 per cent of all squamous celled carcinomas were in male patients, as against 80·5 per cent in males among other histological types. This supports the already well established view that squamous celled carcinoma of the lung is even more a disease of men than is cancer of the lung in general. The adenocarcinomas show a higher proportion of females, but the evidence that this is a real difference in incidence is not very strong. These features are illustrated graphically in Fig. 3.

(vi) *Age and histology.*—There is some indication that squamous celled carcinoma relative to other types attains a maximum incidence in the sixties. In similar comparisons between undifferentiated and differentiated types of tumour the situation is somewhat obscured by lack of uniformity in classification, though in spite of this there do not seem to be any real differences in relative frequency according to age. The variation in the relative incidence of squamous celled carcinoma is seen in Fig. 4.

*Incidence of metastases of all types*

Differences between hospitals in the selection of material for necropsy has presented serious difficulties, but those arising from variations in necropsy findings are even greater. So much seems to depend on pathological techniques, on amount of work in relation to availability of staff, and on the particular

FIG. 3.—Proportionate distribution according to histology compared for males and females.

FIG. 4.—Relative incidence of squamous celled carcinoma found at necropsy for each decade of age.

interests of the pathologists reporting. We propose, therefore, in presenting the information collected from the records to suggest hypotheses rather than to attempt to draw definite inferences.

It has been remarked earlier that a small proportion of the patients had no intracranial examination at necropsy. Therefore in giving statistics relating to intracranial metastases these cases have been excluded from consideration. Indeed it might be argued that in considering the incidence of *all* metastases we should also exclude this group, since patients who did not have an intracranial examination and who had no observable metastases, might, in fact, have had intracranial secondaries. We have, however, considered this (we hope correctly) to be unimportant and have calculated proportions without any such exclusions.

Of the 741 patients, 517 (69·8 per cent) were recorded as having blood-borne metastases at some site or other. The proportions for individual hospitals ranged from 60 per cent to 77 per cent.

(i) *Sex.*—There seems to be no difference in the tendencies for males and females to develop metastases. Secondaries were found in 69 per cent of males and 72 per cent of females.

(ii) *Histology.*—The data confirm the well established fact that squamous celled carcinoma exhibits much less tendency to metastasise than do other types. Metastases were found in association with squamous celled carcinoma at death in only 41 per cent of cases as compared with 79 per cent for other types. In the same way it is found that the adenocarcinomas have less tendency to metastasise than the undifferentiated tumours. Secondaries were found in 70 per cent of patients described as having adenocarcinomas, but in 80·4 per cent of patients said to have undifferentiated tumours. These percentages must, however, be accepted with caution in view of the evident differences in interpretation between hospitals. The figures are summarised in Table III.

TABLE III.—*The Proportions of Patients with Metastases at any Site as found at Necropsy according to Histology of Primary Tumour.*

| Histology. | Total number of patients. | Number with any metastasis. | Per cent with any metastasis. |
|---|---|---|---|
| Undifferentiated | 470 | 378 | 80·4 |
| Adenocarcinoma | 93 | 65 | 69·9 |
| Squamous celled carcinoma | 177 | 73 | 41·2 |
| Type not recorded | 1 | 1 | — |
| All types | 741 | 517 | 69·8 |

(iii) *Age.*—It would appear that the incidence of metastases in general decreases with age. There are, however, three other possible explanations which we must first consider, namely :

(*a*) That the proportion of squamous celled carcinomas increases with age.

(*b*) That older patients who are generally more frail may have a greater tendency to die before metastases become established.

(*c*) That there may be a tendency for less thorough necropsies to be performed on aged patients at some hospitals.

It seems that (*a*) may be discounted at least on the basis of the present data. There is evidence that the proportion of squamous celled tumours is highest in the sixties but none that there is a uniform increase beyond these ages. We cannot dispose of (*b*) so easily, since in the present data no record was made of the duration of the disease. Any further investigation on these lines should certainly take account of this additional factor. We are left with (*c*) and it is conceivable that this may explain the observed decrease in part at least. In examining the incidence of metastases by age for separate hospitals it is quite evident that the extent and nature of the decrease varies. The agreement between hospitals is fairly good at younger ages, but becomes bad at advanced ages. It would seem, therefore, that whether or not tumours in older patients exhibit less tendency to metastasize, the position may be confused by differences between hospitals in their attitude towards the post-mortem examination of aged patients.

(iv) *Sex and histology.*—A consideration of the interaction between these two factors reveals nothing significant.

(v) *Sex and age.*—In Fig. 5*a*, we see how the incidence of metastases is related to age for males and females separately. It would appear from this that females have a lower metastasis rate at ages below 45 but that at ages over 45 the position is reversed. This, together with the fact that the rate for females attains a peak at about 45 years while for males it decreases continually, might suggest the influence of hormone balance on the development of metastases. If, however, we correct for the proportions of different histologies at each age the excess incidence for females largely vanishes, though the male excess at early ages is accentuated (Fig. 5*b*).

FIG. 5.—Incidence of metastases by sex and age.

(vi) *Age and histology.*—The decline in the general metastasis rate with age noted above appears to hold for individual histological types in varying degrees, although the position is confused for the reasons given in (iii) above. All the figures are, however, illustrated in Fig. 6.

From this point we shall adopt a similar scheme in examining the incidence of metastases at particular sites of the body. We have seen that undifferentiated tumours are much more likely to produce metastases than are squamous celled tumours. This may be due to the fact that undifferentiated emboli have a greater overall potentiality for growth than squamous cells. On the other hand it may mean that undifferentiated tumours liberate more emboli than do squamous-celled tumours, but that the emboli which are liberated in either case have a growth potential which, though not inherently very different, may differ according to the host tissues in which they lodge. Under the latter hypothesis it is of interest to consider the incidence of specific metastases from a certain type of tumour expressed as a proportion of the total number of patients with tumours of that type *who were noted as having metastases.* It is proposed to express the incidence in this way whenever histological type is a factor under consideration.

*The incidence of metastases at individual sites.*

There is no doubt that the liver is the organ in which metastases from carcinoma of the bronchus are most commonly found. In this series 39·3 per cent of necropsies revealed secondaries in this organ. It is also worth noting that there was quite good agreement between the various hospitals ; proportions observed ranged from 36 to 44 per cent, except for a figure of 27 per cent in one rather small group.

FIG. 6.—Incidence of metastases according to age and histology.

The only other site for which the incidence of metastases approaches that of the liver is the adrenals. The proportion observed with metastases in these organs is 33·5 per cent. In this case, however, the agreement between hospitals is not so good and extends from 25 to 47 per cent. When we consider the relative sizes of liver and adrenals, this high rate is seen to be a matter of outstanding interest.

Next in order of relative frequency are brain metastases. We are obliged to base the incidence at this site on those cases in which an intra-cranial examination was performed. The incidence in this group is 25·7 per cent, with figures for individual hospitals extending from 17 to 43 per cent. A special selection process operates at the latter hospital where there is considerable interest in neuro-surgery. It is indeed a remarkable fact that many patients attending this hospital on account of neurological symptoms alone are subsequently found at necropsy to have a primary tumour in the lung.

Metastases in kidneys were recorded in 15·4 per cent of necropsies with figures for individual hospitals ranging from 12 to 25 per cent.

The overall incidence of bone metastases in the series is 14·6 per cent, but this has little import when we observe that the incidence for the separate hospitals is widely dissimilar (6 per cent to 27 per cent) and general, detailed skeletal examination uncommon. It is clearly futile to attempt to draw any conclusions from data relating to bone metastases unless very detailed necropsies were performed with this object in view.

The only other organ in which metastases occur to an appreciable extent is the pancreas. In the whole series the pancreas was recorded as being invaded in 11·6 per cent of cases with a range of 7 to 27 per cent between the hospitals.

Other sites of some interest are the spleen and the thyroid, but the incidence of metastases recorded for these organs is rather small being 5·3 and 3·6 per cent respectively.

The only other organs which will subsequently be referred to specifically are the ovaries. Ovarian metastases were noted in seven patients, i.e. 5·4 per cent of females in the series.

Other sites for which metastases have been recorded include the pituitary (7), the prostate (3), the testis (1), the alimentary tract (37), the meninges (11), the heart (17), the medulla (1), the uterus (1), and skin (12). These will not be referred to again here either because of their rarity or because of an evident lack of uniformity in the frequency with which they are reported by different hospitals. Table IV gives the figures for the overall incidence of specific metastases together with the range of values provided by the data from the various hospitals.

TABLE IV.—*The Observed Incidence of Metastases at Various Sites*

| Site. | Number of metastases reported. | Overall incidence (%). | Range (%). | |
|---|---|---|---|---|
| Liver | 291 | 39·3 | 27 | 44 |
| Adrenals | 247 | 33·5 | 25 | 47 |
| Brain* (excluding meninges) | 166 | 25·7 | 17 | 43 |
| Kidney | 114 | 15·4 | 12 | 25 |
| Bones | 108 | 14·6 | 8 | 27 |
| Pancreas | 86 | 11·6 | 7 | 27 |
| Spleen | 39 | 5·3 | 2 | 10 |
| Thyroid | 27 | 3·6 | 1 | 11 |
| Ovary† | 7 | 5·4 | | |

* Percentage of necropsies at which an intracranial examination was made.
† Percentage of females only.

(i) *Sex.*—The two sexes differ in only one remarkable way in regard to the incidence of metastases at individual sites. This may be due to the fact that in basing metastasis rates on the whole data we are missing important features. We shall, however, examine the effect of age and histology jointly with sex in subsequent sections. At most sites there is a slightly higher rate of metastasis for females. This may be due in part at least to the less frequent occurrence of squamous celled tumours among females.

The adrenals form the one exception, since here the metastasis rate for males is higher than for females, and although this difference is not significant for the whole data taken together it does, as we shall see, assume greater importance when age is also considered. The percentage incidence for metastases is shown by site for males and females in Table V.

(ii) *Histology.*—The same incidence gradient from undifferentiated to squamous is seen for metastases at particular sites as for all metastases taken together. The gradient is, however, considerably modified in the case of secondaries in the kidney. It is, perhaps, more instructive at this stage to consider incidence based not on all necropsies but only on those revealing metastases at any site. We shall then be answering the question : " given that metastases have occurred, is there any difference in the manner in which they are distributed through the body for primary tumours of different histology ? "

TABLE V.—*The Incidence of Metastases at Various Sites according to Sex*

| Site. | Males. Number with metastases. | Males. Per cent. | Females. Number with metastases. | Females. Per cent. |
|---|---|---|---|---|
| Liver | 235 | 38·5 | 56 | 43·1 |
| Adrenals | 212 | 34·7 | 36 | 27·7 |
| Brain* (excluding meninges) | 138 | 25·7 | 28 | 25·5 |
| Kidneys | 90 | 14·7 | 24 | 18·5 |
| Bones | 90 | 14·7 | 18 | 13·8 |
| Pancreas | 66 | 10·8 | 20 | 15·4 |
| Spleen | 28 | 4·6 | 11 | 8·5 |
| Thyroid | 21 | 3·4 | 6 | 4·6 |
| All organs | 424 | 69·4 | 93 | 71·5 |

*Based on 537 males and 110 females who were examined intracranially at necropsy.

When we examine these modified metastasis rates we find that there is much more agreement between different histologies for metastases at most sites, e.g. liver, adrenal, spleen, thyroid and bones. This procedure does, however, emphasize the differences for kidney and pancreas. In the case of kidney the rate for undifferentiated tumours is relatively low, while for pancreas the corresponding rate is still rather greater than for the other types. These features are illustrated in Fig. 7.

FIG. 7.—Proportions of patients with metastases who had metastases in certain specified organs.

(iii) *Age.*—The tendency for metastases to become less common with advancing age is, in general, preserved for metastases in individual organs, though there is usually some modification in the overall pattern We are also at the mercy of the difficulties referred to under the heading Incidence of Metastases of all Types, Section (iii).

The incidence of liver metastases seems to remain fairly constant at about 39 per cent throughout the age range, though there is a possibility that it is higher in the forties (48 per cent).

The position relating to brain secondaries is not very clear. The overall incidence is found to be 25·7 per cent, but the variation about this figure is considerable at individual hospitals. Furthermore, the variation of incidence with age does not follow a consistent trend at different hospitals. The data of two hospitals point to a fairly level incidence of about 30 per cent, while at another the picture is biased by neurosurgical interests. The remainder indicates an incidence declining from 46 per cent at ages under 50, to 12 per cent in old age. Brain metastases are dealt with further under the heading Brain Metastases from Bronchial Carcinoma.

FIG. 8.—The incidence of metastases at various sites according to age and sex.

Adrenal metastases occur very commonly at ages under 40 years (61 per cent) but see (v) below.

In regard to ovarian metastases it seems important that five out of the seven instances recorded occurred in patients less than fifty years of age.

(iv) *Sex and histology.*—Nothing significant emerges from a consideration of these two factors jointly.

(v) *Sex and age.*—The high incidence of adrenal metastases in patients dying at an early age was found to be particularly characteristic of males. Fourteen out of 17 (82 per cent) necropsies performed on males under 40 years of age revealed adrenal metastases. Fig. 8 shows in detail the incidence of metastases at various sites according to sex and age.

(vi) *Histology and age.*—Here again we treat incidence as based on those patients in whom some metastasis was found, i.e. relative incidence. This analysis brings out once more the higher relative incidence of kidney metastases from adenocarcinomas and squamous celled tumours as compared with undifferentiated tumours, particularly at ages over 50.

(vii) *Mechanical factors.*—It is of interest to see whether the incidence of metastases in particular organs is related in any way to such basic factors as weight of tissue, total blood flow through the organ in unit time and total blood flow in unit time per gramme of tissue. The relevant figures are set out in Table VI. It will be seen that if there is any relationship it must be a very complex one, doubtless involving other variables. There might conceivably be some direct dependence on the weight of the organ, since both liver and brain show such high incidence. This alone would leave unexplained, however, the frequent occurrence of adrenal secondaries, not to mention the infrequent occurrence of secondaries in such an extensive organ as the skin, although the post-mortem fading of these secondaries is well recognised.

Any suggestion that the incidence of metastases might be inversely related to the velocity of blood flow is readily countered by the example of the spleen through which the blood flows much more slowly than through the brain. Furthermore, the extent of the capillary bed does not seem to exert a marked influence, since the skin and skeletal muscles are rarely affected.

TABLE VI.—*Frequency of Metastases from Carcinoma of the Bronchus Related to Weight of and Blood Flow in Various Organs*

| Organ. | Percentage of all patients having metastases in organ. | Weight of organ in grammes.* | Blood flow per 100 g. of tissue in c.c. per minute.† | Approximate blood flow in the whole organ in c.c. per minute. |
|---|---|---|---|---|
| Adrenals | 33·5 | 12 | 600–700 | 80 |
| Thyroid | 3·6 | 40 | 560 | 220 |
| Pancreas | 11·6 | 110 | — | — |
| Spleen | 5·3 | 155 | 40 | 60 |
| Kidneys | 15·4 | 300 | 400 | 1300 |
| Brain | 25·7 | M 1400<br>F 1275 | 200 | 2800 |
| Liver | 39·3 | 1650 | 50<br>(arterial) | 800 |

* Sunderman and Boerner (1950).

† Best and Taylor (1950).

Since the elementary haemodynamic processes seem to have no simple bearing on the distribution of metastases from carcinoma of the bronchus some more subtle differences in capillary bed structure or in by-passing mechanisms must be invoked if the mechanical theories are to be upheld. Detailed work on this aspect of the problem in man is required and might perhaps come from the use of micro-radiographic techniques similar to those employed by Barclay (1951).

The " seed and soil " aspect of this problem is likely to be concerned with an interaction between the structure and biochemical composition of the host tissues, on the one hand, and the histological characteristics of the emboli, on the other.

*Brain metastases from bronchial carcinoma*

This subject has aroused much interest for some years and for this reason the brain metastases recorded in connection with the present data are being made the subject of a separate article.

The proportion of necropsies revealing brain metastases, namely 25·7 per cent, appears to be consistent with the results of similar investigations over the past 40 years.

It is shown that brain metastases tend to be associated with metastases in other organs but that the extent of this association varies for different organs, e.g. the association with liver metastases is low but high with metastases in the pancreas. It is suggested that these differences may arise merely from relative lethality.

It is also shown that brain metastases associated with metastases elsewhere in the body have a greater tendency to be multifocal.

The proportion of single brain metastases found to be located in the cerebellum (38 per cent) does not in itself suggest any more interesting hypothesis than a direct relationship to tissue mass.

*Conclusions*

(i) A study of the incidence of metastases in selected organs in relation to the haemodynamic normal values associated with them reveals no simple relationship though there does appear to be some correlation between the occurrence of metastases and the weights of organs, with the adrenals as the outstanding exception.

(ii) The high incidence of adrenal metastases in young males, together with the fact that the few instances of ovarian metastases were found mainly at ages under 50, might suggest a relationship between metastasis rate and biological activity in these endocrine organs at least.

(iii) The proportion of adenocarcinomas classified as undifferentiated tumours of the bronchus varies greatly among different pathologists. The proportion of undifferentiated tumours and adenocarcinomas taken together which were classified as adenocarcinomas varied from 6 to 31 per cent in this series. Particularly careful and critical examination is therefore required in drawing conclusions regarding different aetiologies in groups with different histologies.

(iv) It would seem likely that the incidence of metastases and their distribution depends on a complicated balance between the following factors : degree of tumour cell differentiation, state of the tissues of the tumour bed, number of cells liberated, size of adhering clumps, size of organs, rate of blood flow at different sites, extent and nature of capillary beds and the metabolic activity of different tissues. There is still too little data available on many of these points. There are however, enough exceptions (for example adrenals to dependence on organ size, kidneys to dependence on degree of tumour cell differentiation, spleen to dependence on rate of blood flow) to many of the more simple factors to make it highly improbable that any purely mechanical theory could alone be held responsible for metastases. A detailed study of the more obvious exceptions to the simple theories might provide useful information about the factors determining the incidence and distribution of blood-borne metastases, that is to say the main factors which are at present delaying further advance in the effectiveness of cancer treatment.

SUMMARY

1. The records of 741 necropsies relating to carcinoma of the bronchus drawn from eight London Hospitals (four teaching and four Regional Board hospitals) have been analysed.

2. These necropsies were taken from a total of 10,430 performed at these hospitals in the years 1948–52. In the hospitals which do not specialize in malignant disease the proportion of all necropsies relating to lung cancer approaches 7 per cent.

3. The proportion of females in this material was 17·5 per cent, corresponding to a ratio of 4·7 males to 1 female.

4. The mean age at death of the patients was about 57 years in the Teaching Hospitals and about 64 years in the Regional Board Hospitals.

5. The greater proportion of necropsies, 63·5 per cent, revealed undifferentiated carcinoma ; 23·9 per cent revealed squamous celled carcinoma and 12·6 per cent adenocarcinoma. Differences of interpretation between hospitals may, however, affect the validity of these figures, particularly the last.

6. There was a somewhat higher proportion of squamous celled carcinoma among males.

7. In 69·8 per cent of cases metastases were found at some site or other. For individual histological types the corresponding figures are : undifferentiated carcinoma 80·4 per cent, adenocarcinoma 70 per cent, squamous celled carcinoma 41 per cent.

8. An apparent tendency was found for the metastasis rate to decline with age, probably due, in part, to some differences in attitude towards the post-mortem examination of aged patients.

9. The most common sites for metastasis were found to be liver (39·3 per cent), adrenals (33·5 per cent) and brain (25·7 per cent), though only in the case of the liver was there good agreement between hospitals.

10. The only marked sex-difference in the incidence of metastases was found in the higher incidence of adrenal metastases among males dying at ages under 40 years. Out of seventeen such cases, the adrenals were found to be involved in fourteen.

We are especially grateful to a number of pathologists who most generously have allowed us free access to their necropsy records and permission to publish the results of our study of them : they were Professor T. Crawford of St. George's Hospital, Professor J. H. Dibble of Hammersmith Hospital, Professor R. W. Scarff of Middlesex Hospital, Dr. A. B. Bratton and Dr. C. C. Bryson of the Whittington Hospital, Dr. A. G. Signy and Dr. M. Gillespie of St. Mary Abbots Hospital and Dr. J. W. Whittick of the Royal Marsden Hospital.

We are indebted to Professor D. W. Smithers who both suggested this investigation to us and has given us much valuable assistance in the preparation of the text.

While acknowledging this help and kindness we must make it clear that they cannot be held in any way responsible for the comments we have made on the data collected.

*Riassunto :* La distribuzione e la crescita delle metastasi ematiche dei tumori appare, secondo le odierne conoscenze, dipendere da una complessa associazione tra fattori meccanici e biochimici.

Poichè il carcinoma bronchiale dell'uomo offre, per la stessa sua sede, il miglior materiale di studio del problema, gli AA. hanno scelto questo tipo di tumore e ne hanno studiato la distribuzione delle metastasi ematiche in 741 casi tratti da 10·430 autopsie eseguite in 8 Ospedali di Londra dal 1948 al 1952.

Le principali osservazioni derivanti sono : (a) la conferma che i vari tipi istologici esibiscono una diversa attitudine a metastatizzare ; (b) che la tendenza alla diffusione metastatica diminuisce con l'aumentare dell'età ; (c) che vi è una tendenza a metastasi associate tra certi organi ; (d) che la distribuzione delle metastasi nei vari organi si dimostra soggetta ad una interazione tra semplici processi emodinamici, caratteristiche istologiche degli emboli neoplastici e biochimismo del tessuto ospitante.

La elevata incidenza di metastasi surrenali nei maschi di età relativamente giovane insieme al fatto che gran parte dei pochi esempi di metastasi ovariche nelle donne sono state trovate ad età inferiori ai 50 anni, può suggerire una relazione tra possibilità di impianto delle metastasi e attività biologica di questi organi.

### REFERENCES

ANDERSON, R.—(1951) *J. Neurosurg.*, **8**, 411.
BARCLAY, A. E.—(1951) 'Micro-arteriography ; and other Radiological Techniques Employed in Biological Research.' Oxford (Blackwell Scientific Publications).
BATSON, O. V.—(1940) *Ann. Surg.*, **112**, 138.—(1942) *Amer. J. Roentgenol.*, **48**, 715.
BEST, C. H. AND TAYLOR, N. B.—(1950) 'The Physiological Basis of Medical Practice.' 5th Ed. London (Ballière, Tindall and Cox).
BRITISH EMPIRE CANCER CAMPAIGN.—(1950) *Ann. Rep.* **28**, 97.
COMAN, D. R., DE LONG, R. P. AND MCCUTCHEON, M.—(1951) *Cancer Res.*, **11**, 648.
DE LONG, R. P. AND COMAN, D. R.—(1950) *Ibid.*, **10**, 513.
HEADY, J. A. AND KENNAWAY, E. L.—(1949) *Brit. J. Cancer*, **3**, 311.
KREYBERG, L. AND POPPE, E.—(1940) *Lancet*, i, 593.
LUCKE, B., BREEDIS, C., WOO, Z. P., BERWICK, L. AND NOWELL, P.—(1952) *Cancer, Res.*, **12**, 734.
REGISTRAR-GENERAL (1950, 1951, 1952, 1953) *Statistical Reviews of England and Wales for the Years* 1948 *to* 1952. Tables Part I. Medical. London (H.M. Stationery Office).
STEINER, P. E., BUTT, E. M. AND EDMONDSON, H. A.—(1950) *J. nat. Cancer Inst.*, **11**, 497.
SUGARBAKER, E. D.—(1952) *Cancer*, **5**, 606.
SUNDERMAN, F. W. AND BOERNER, F.—(1950) 'Normal Values in Clinical Medicine.' Philadelphia and London (W. B. Saunders Company).
TRUETA, J., BARCLAY, A. E., DANIEL, P. M., FRANKLIN, K. J. AND PRITCHARD, M. M. L.—(1947) 'Studies of the Renal Circulation', p. 127, Footnote 1. Oxford (Blackwell Scientific Publications).
WATANABE, S.—(1954) *Cancer*, **7**, 215.
WILLIS, R. A.—(1952) 'The Spread of Tumours in the Human Body.' 2nd Ed. London (Butterworth).
YOUNG, J. S. AND GRIFFITH, H. D.—(1950) *J. Path. Bact.*, **62**, 293.
ZEIDMAN, I. AND BUSS, J. M.—(1952) *Cancer Res.*, **12**, 731.
*Idem*, MCCUTCHEON, M. AND COMAN, D. R.—(1950), *Ibid.*, **10**, 357.


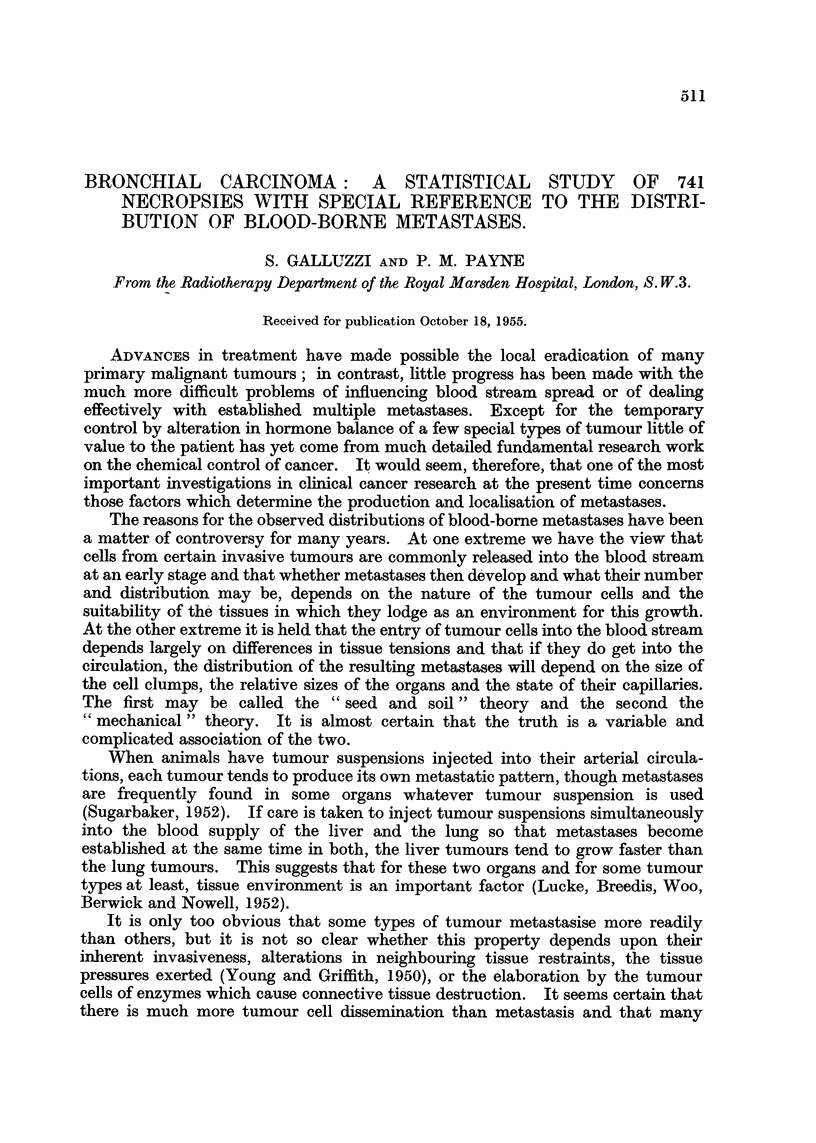

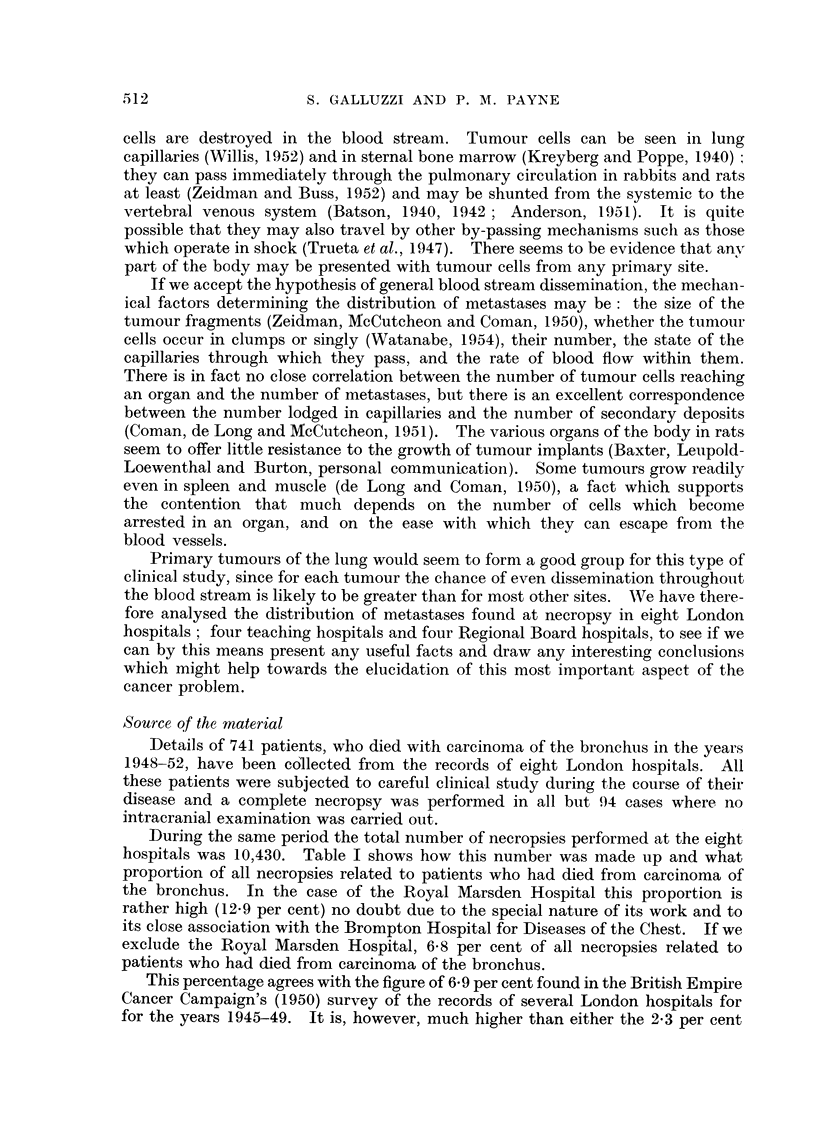

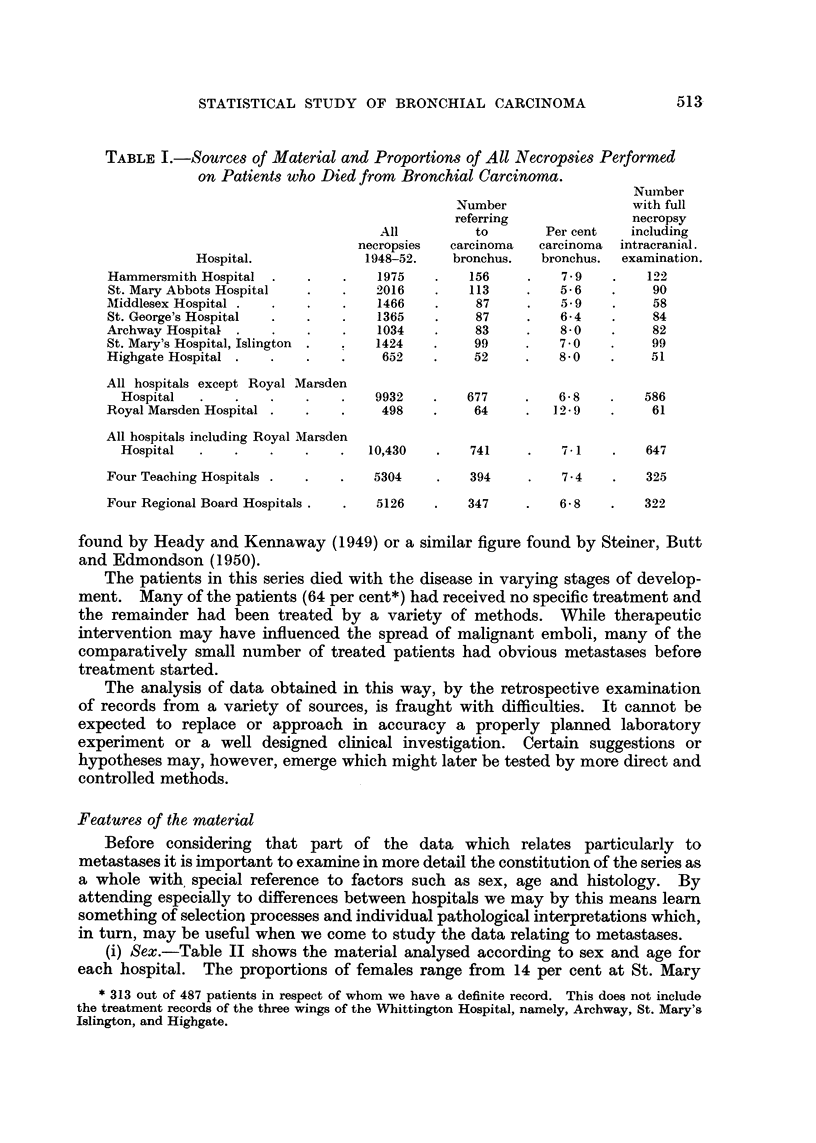

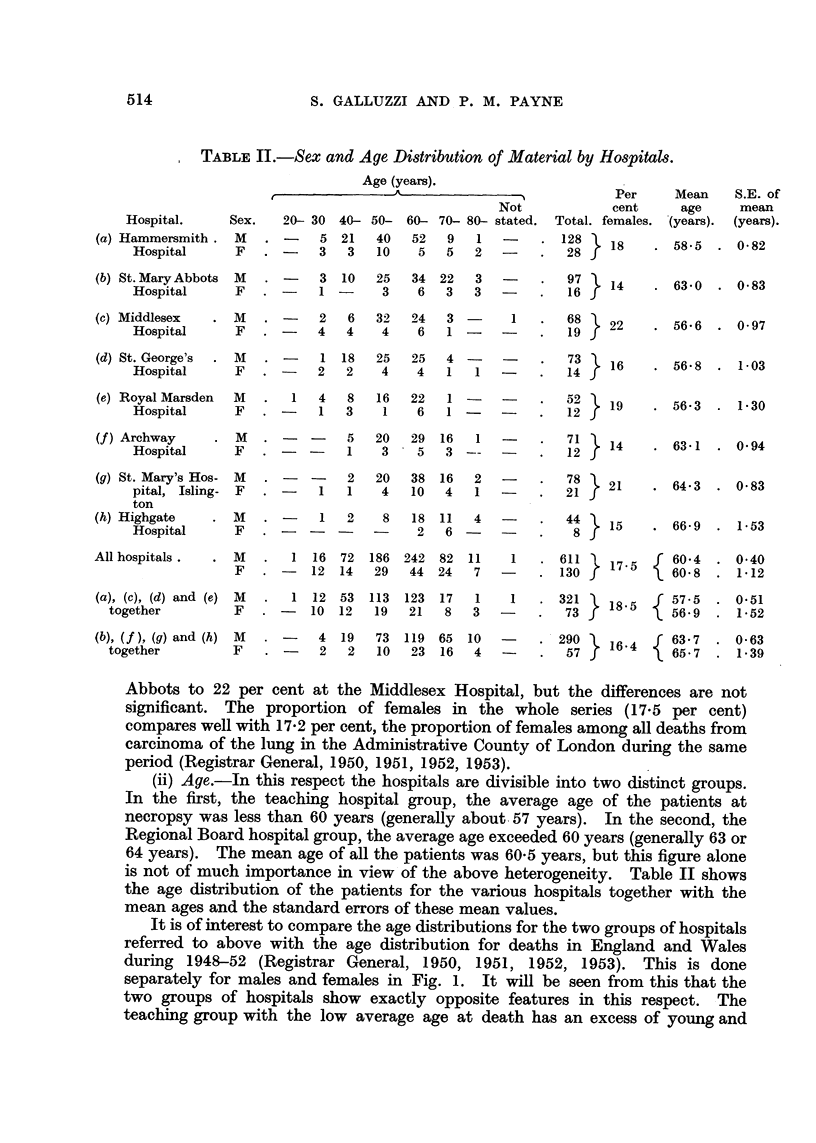

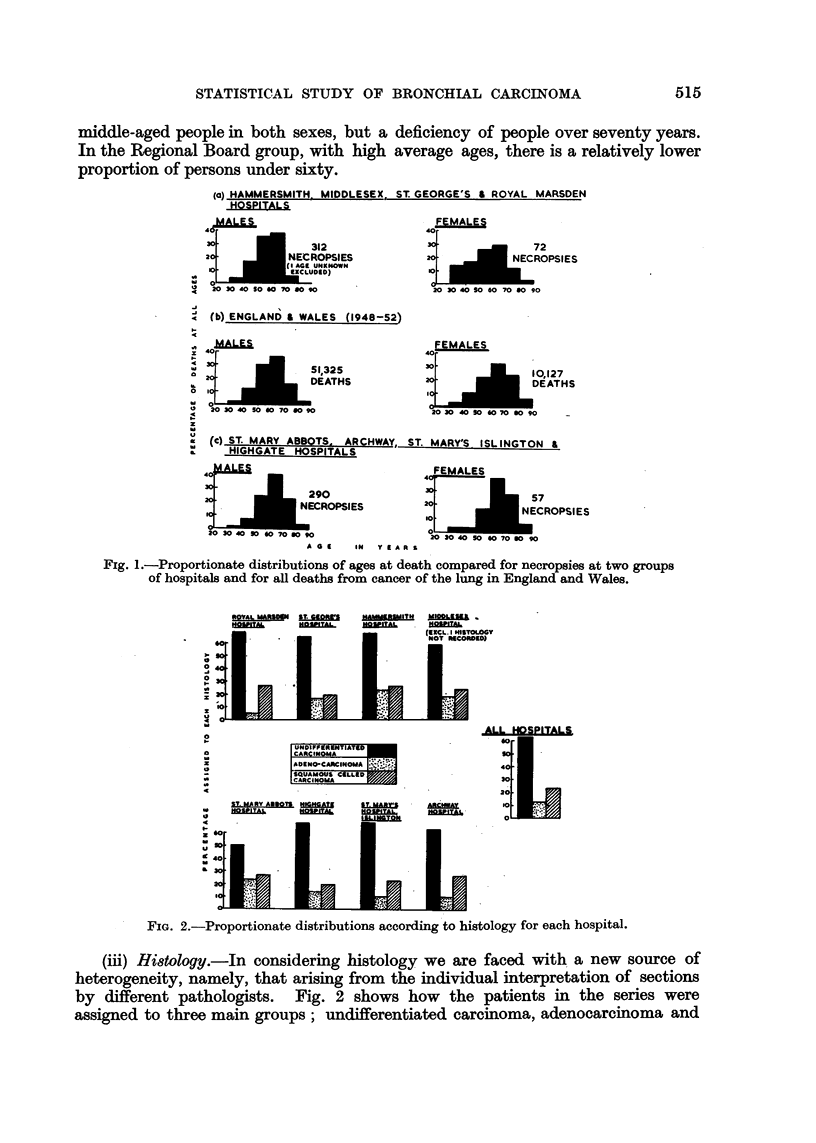

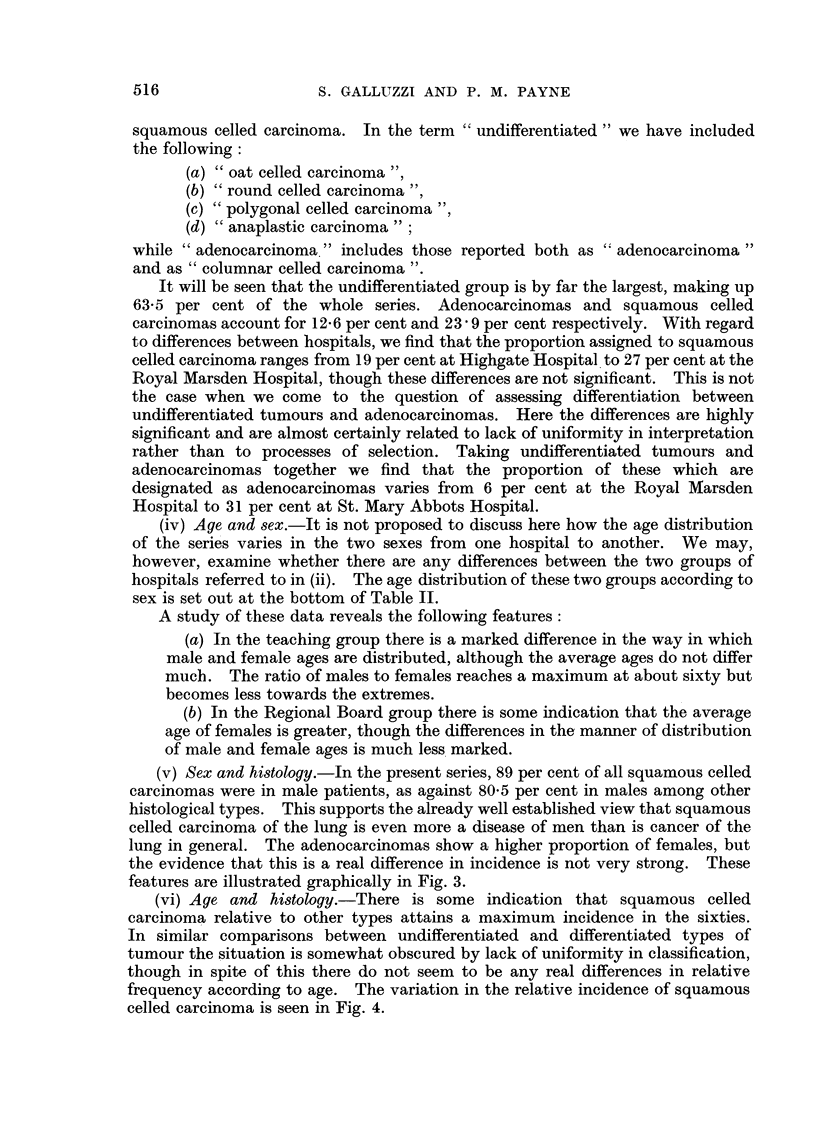

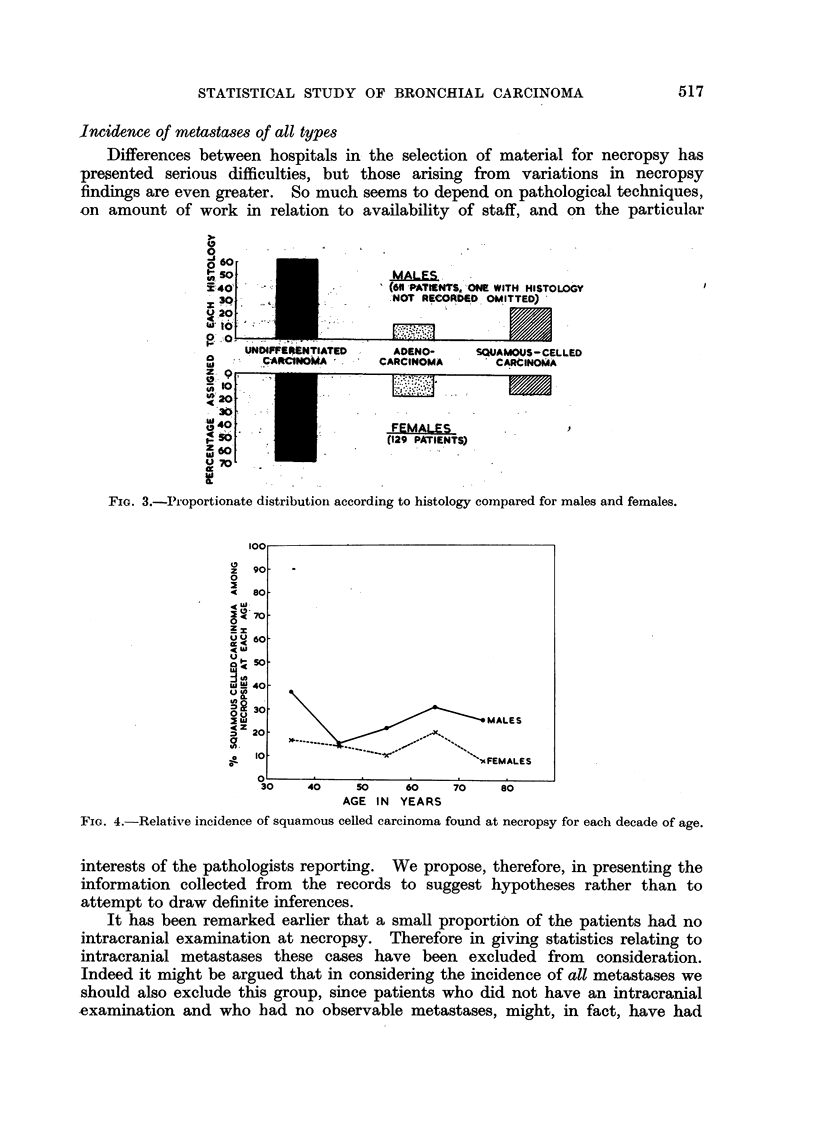

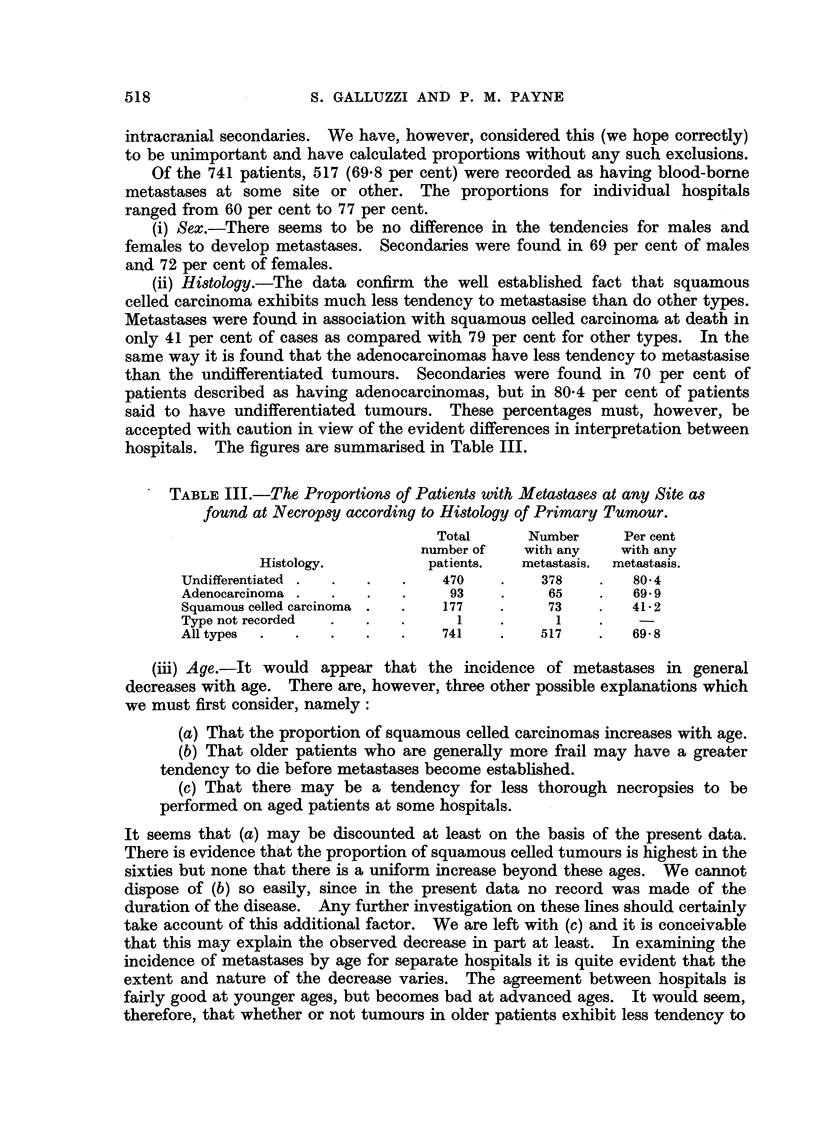

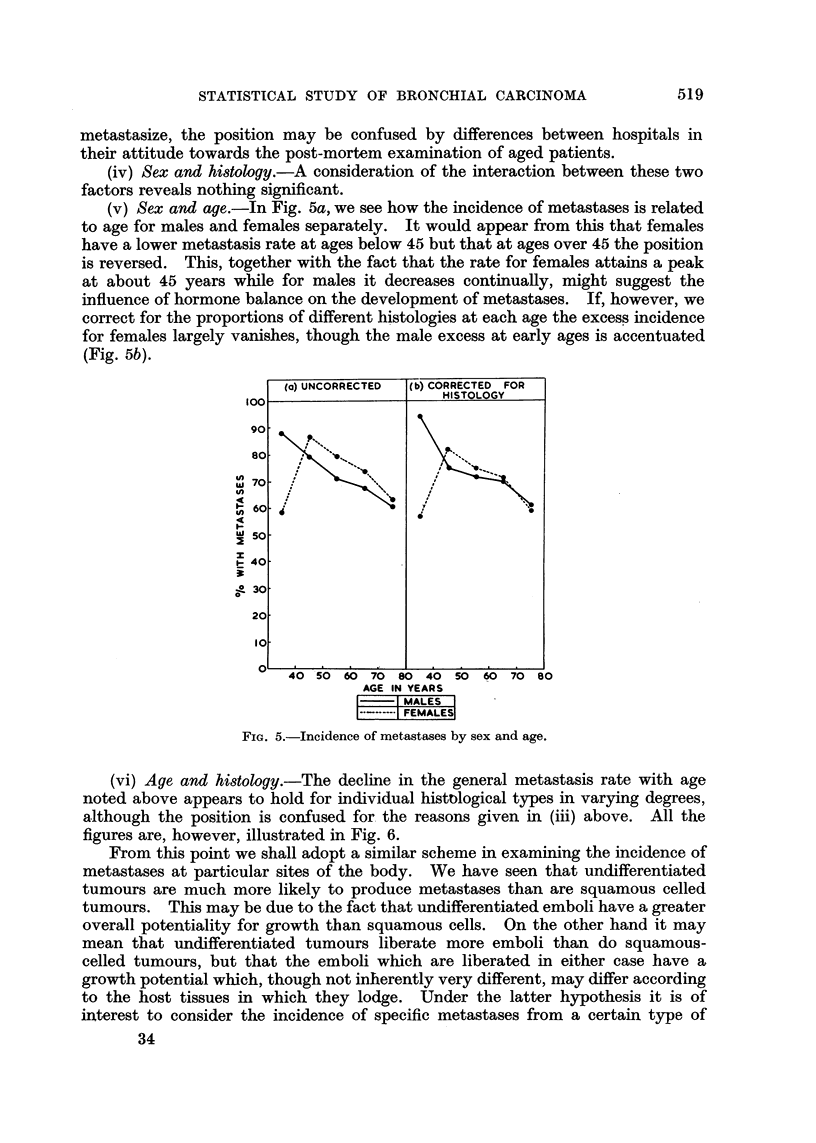

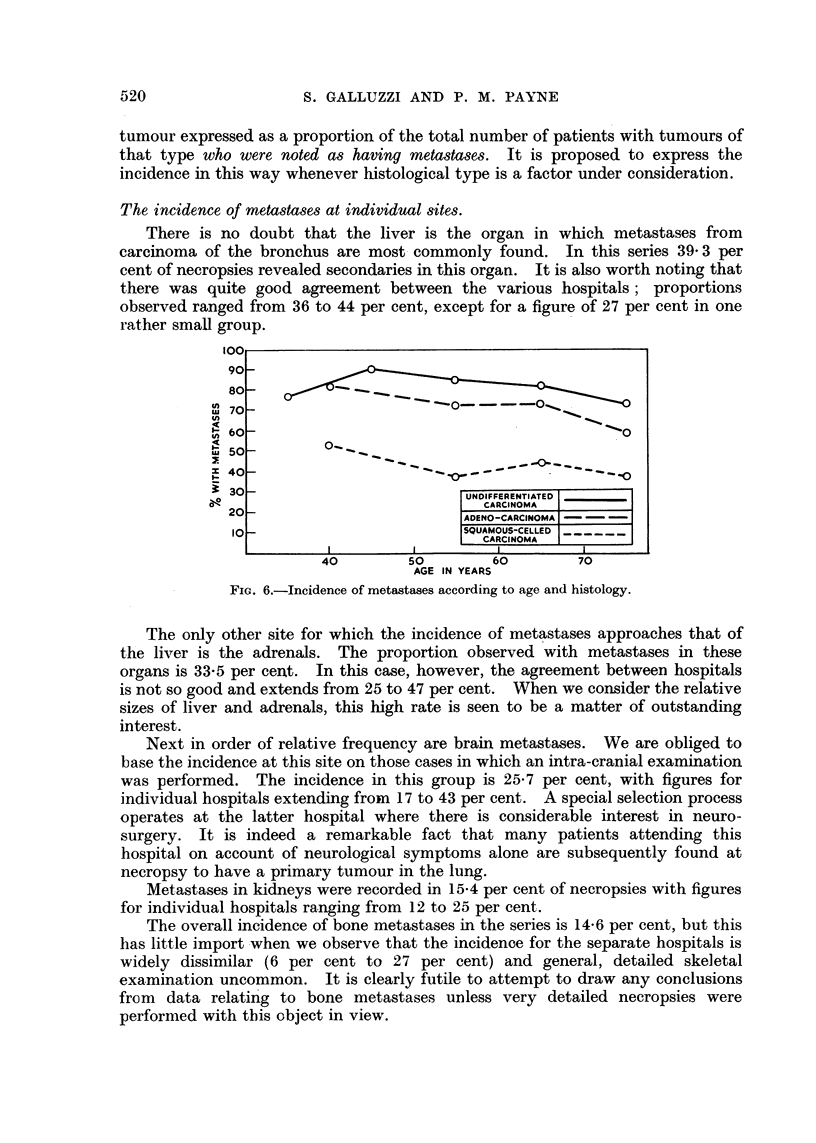

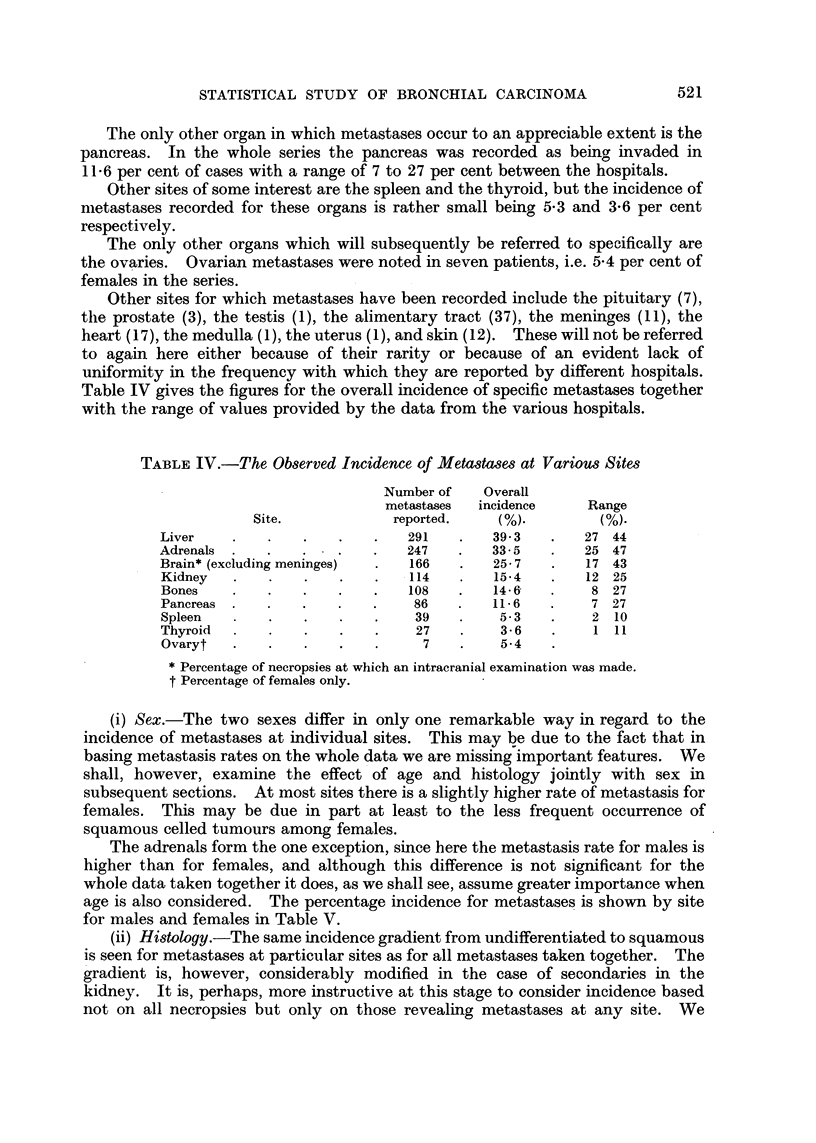

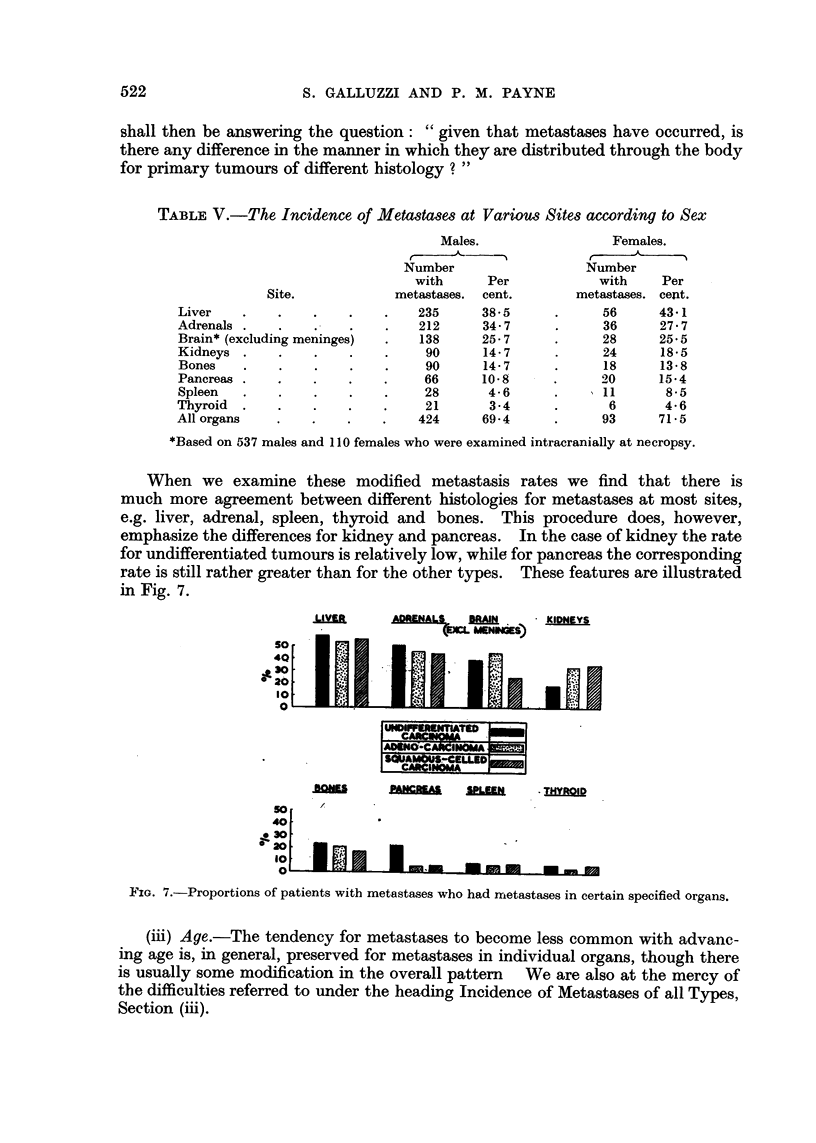

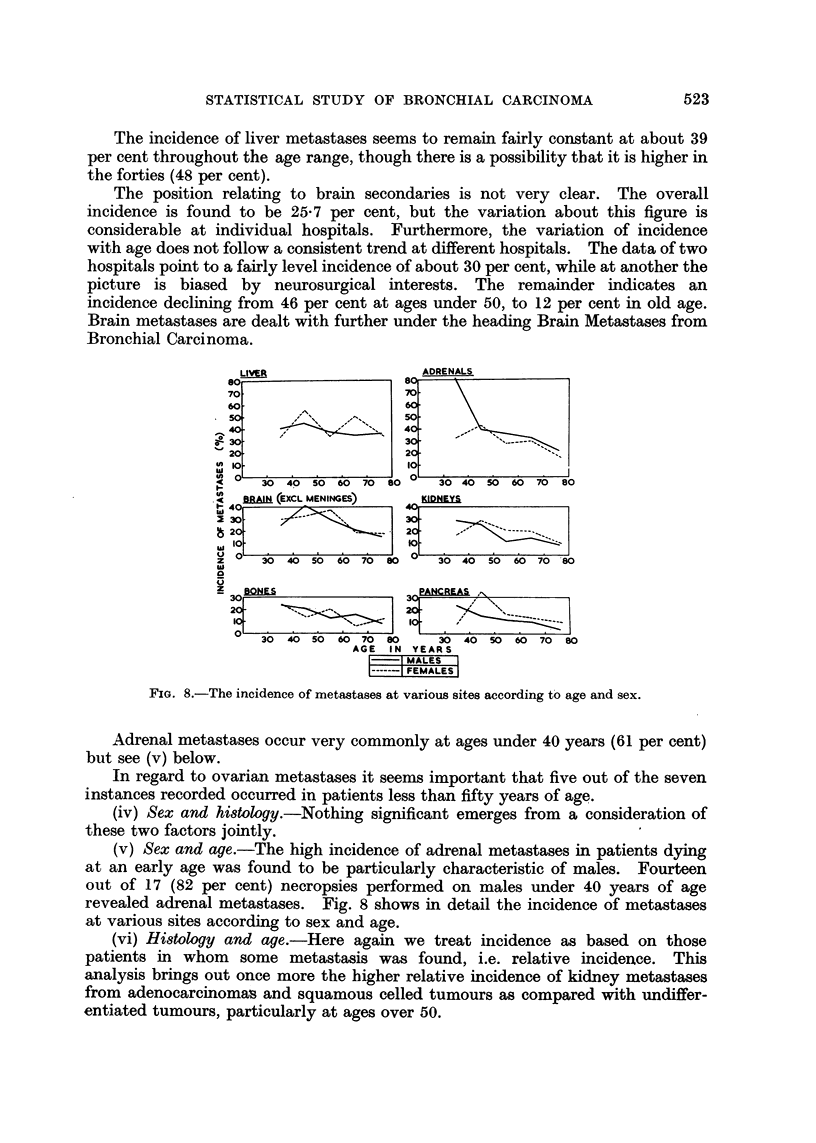

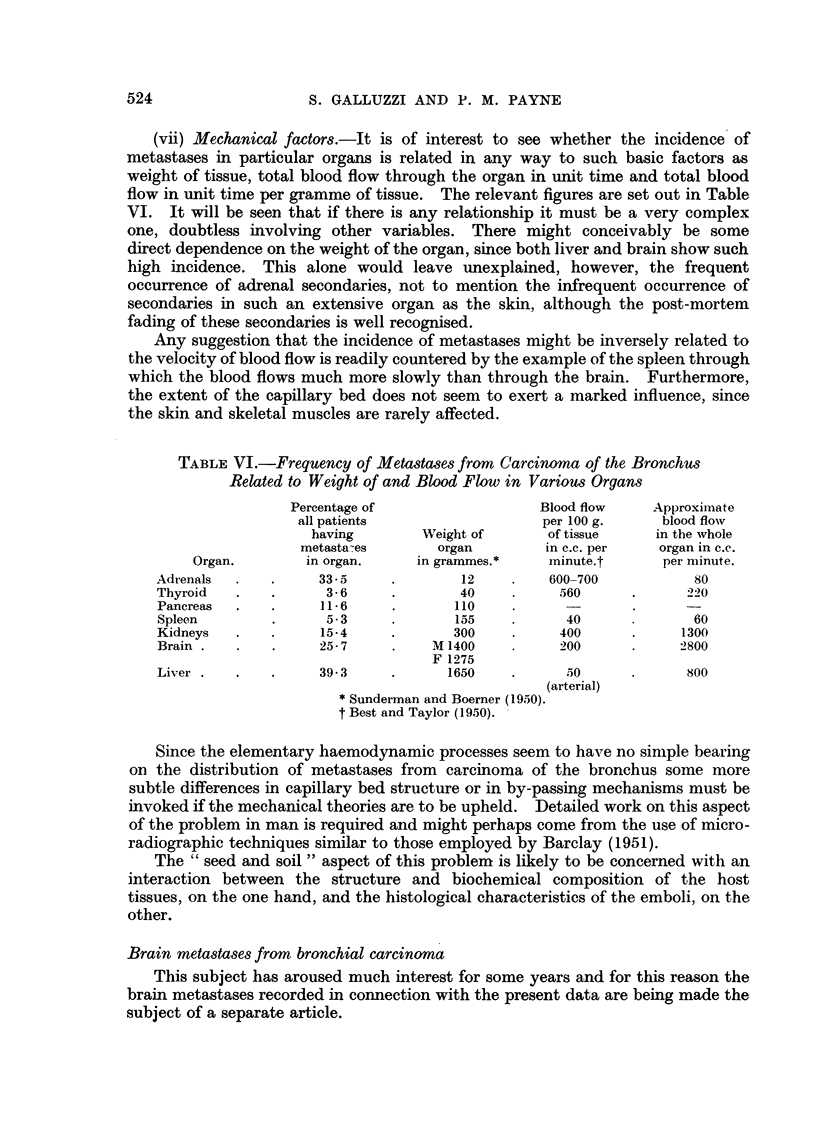

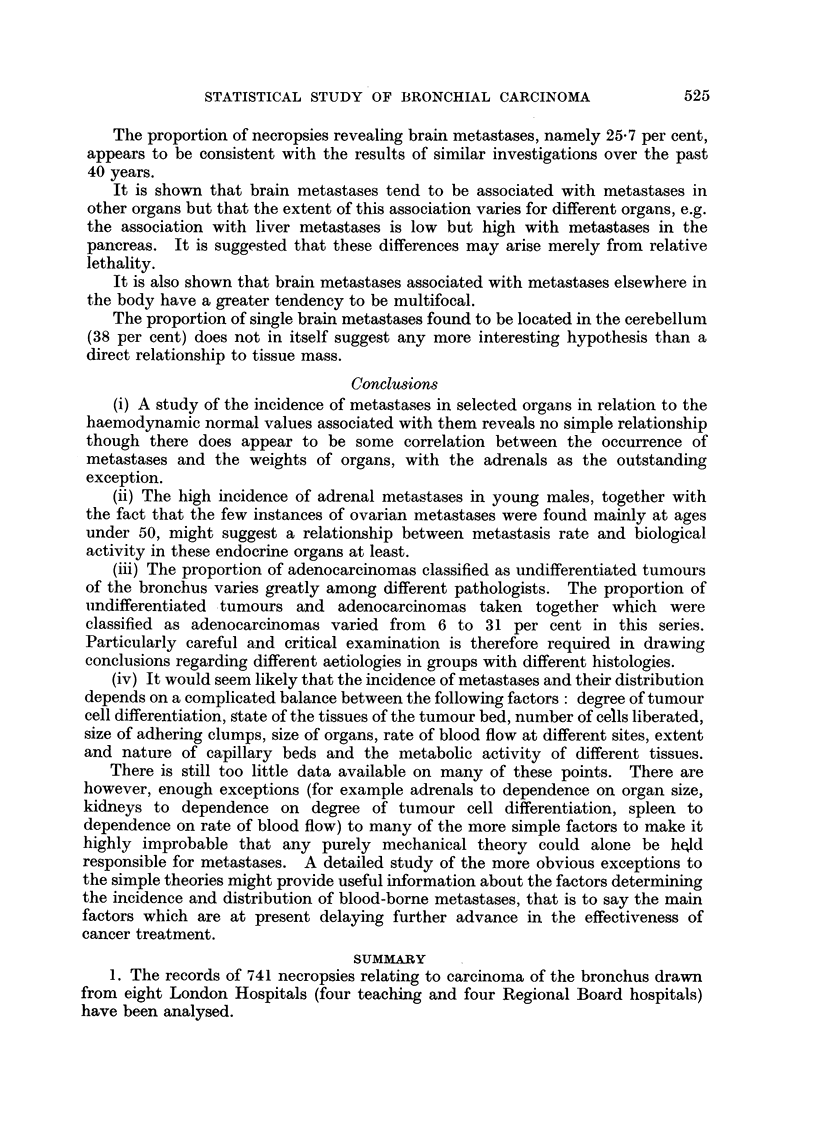

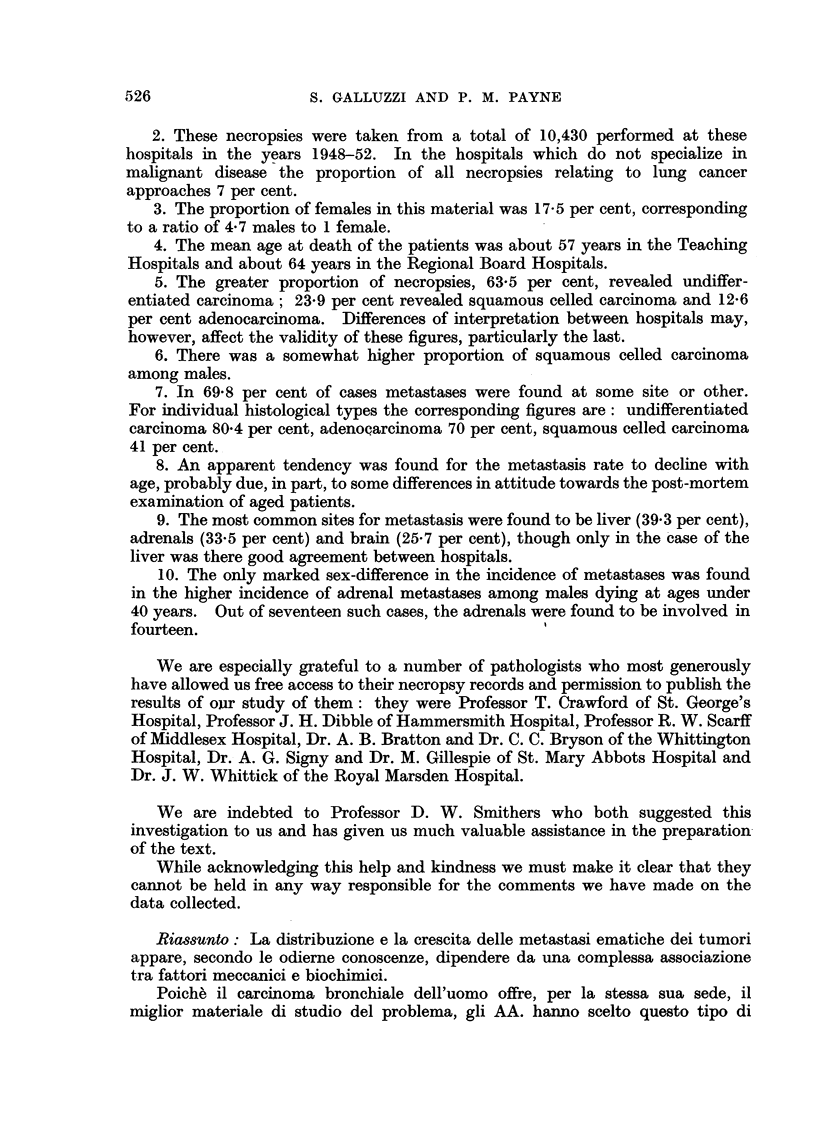

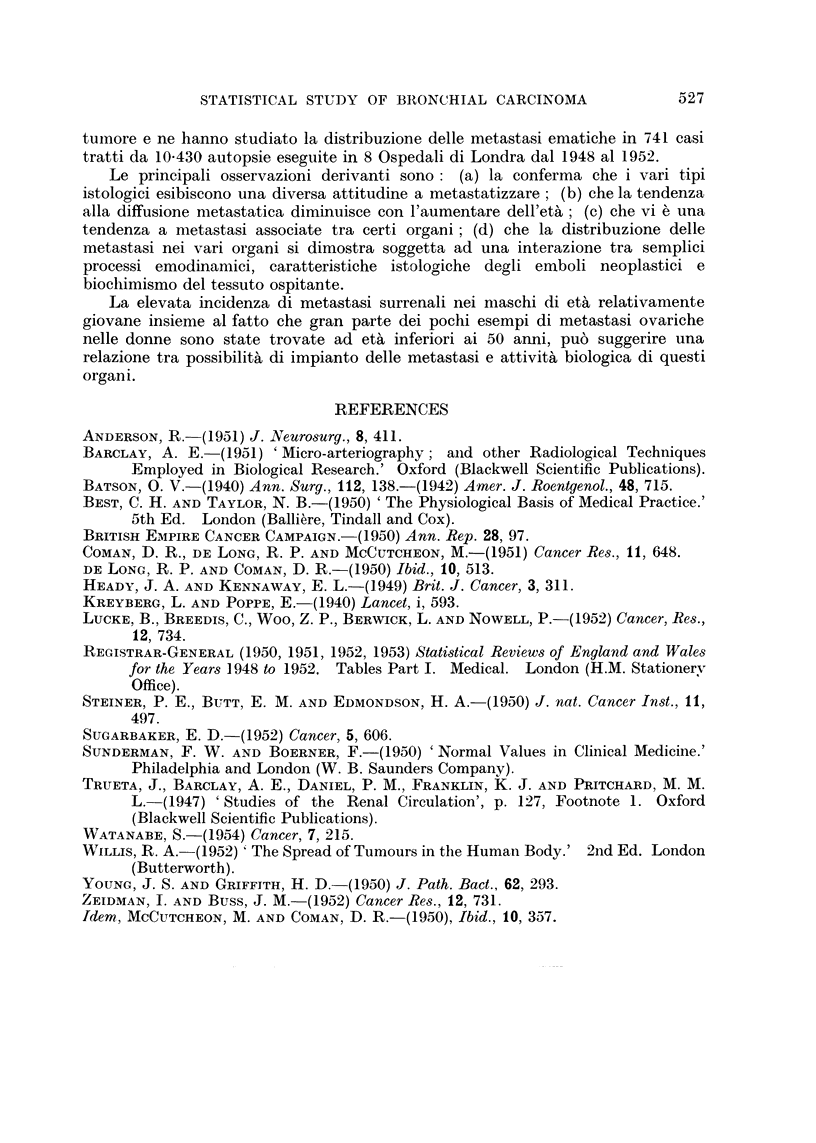


## References

[OCR_01250] ANDERSON R. (1951). Diodrast studies of the vertebral and cranial venous systems to show their probable role in cerebral metastases.. J Neurosurg.

[OCR_01255] Batson O. V. (1940). THE FUNCTION OF THE VERTEBRAL VEINS AND THEIR ROLE IN THE SPREAD OF METASTASES.. Ann Surg.

[OCR_01264] COMAN D. R., deLONG R. P., MccUTCHEON M. (1951). Studies on the mechanisms of metastasis; the distribution of tumors in various organs in relation to the distribution of arterial emboli.. Cancer Res.

[OCR_01266] HEADY J. A., KENNAWAY E. L. (1949). The increase in deaths attributed to cancer of the lung.. Br J Cancer.

[OCR_01271] LUCKE B., BREEDIS C., WOO Z. P., BERWICK L., NOWELL P. (1952). Differential growth of metastatic tumors in liver and lung; experiments with rabbit V2 carcinoma.. Cancer Res.

[OCR_01282] SUGARBAKER E. D. (1952). The organ selectivity of experimentally induced metastases in rats.. Cancer.

[OCR_01298] YOUNG J. S., GRIFFITH H. D. (1950). The dynamics of parenchymatous embolism in relation to the dissemination of malignant tumours.. J Pathol Bacteriol.

[OCR_01299] ZEIDMAN I., BUSS J. M. (1952). Transpulmonary passage of tumor cell emboli.. Cancer Res.

[OCR_01301] ZEIDMAN I., McCUTCHEON M., COMAN D. R. (1950). Factors affecting the number of tumor metastases; experiments with a transplantable mouse tumor.. Cancer Res.

